# Improving interpreting for dementia assessments: Results from the MINDSET trial

**DOI:** 10.1002/alz.085823

**Published:** 2025-01-03

**Authors:** Bianca Brijnath, Marina G. Cavuoto, Simona Markusevska, Andrew Gilbert, Josefine Antoniades, Erika Gonzalez Garcia, Jim Hlavac, Lee‐Fay Low, Dina LoGiudice, Robyn Woodward‐Kron, Joanne Enticott, Kerry Hwang, Jennifer White, Xiaoping Lin

**Affiliations:** ^1^ University of Western Australia, Perth, Western Australia Australia; ^2^ National Ageing Research Institute, Melbourne, VIC Australia; ^3^ University of Melbourne, Melbourne, VIC Australia; ^4^ National Ageing Research Institute, Parkville, VIC Australia; ^5^ Monash University, Melbourne, VIC Australia; ^6^ RMIT University, Melbourne, VIC Australia; ^7^ The University of Sydney, Sydney, NSW Australia; ^8^ The University of Melbourne, Melbourne, VIC Australia; ^9^ University of Newcastle, Newcastle, NSW Australia

## Abstract

**Background:**

The Improving Interpreting for Dementia Assessments (MINDSET) study aimed to upskill interpreters through an online co‐designed course in dementia and cognitive assessments.

**Methods:**

A single‐blinded randomized controlled digital trial conducted between June 2022 and November 2023. Interpreters were randomized to training or waitlist control conditions with 3‐ and 6‐month follow‐up. The primary outcome was a composite *Z‐*score comprising dementia and cross‐cultural knowledge, translation and ethical knowledge, and observed interpreting skills. Preliminary analyses were conducted using a mixed ANOVA with assessment period as the within‐subjects factor and intervention group as the between‐subjects factor, controlling for age.

**Results:**

126 interpreters (M*age =* 44.13 years (*SD* = 12.71) completed baseline (22 Arabic, 14 Cantonese, 6 Greek, 14 Italian, 64 Mandarin, 6 Vietnamese), 3m follow‐up (n = 100) and 6m follow‐up (n = 101). For the primary outcome, there were no significant main effects for assessment period *F* (2, 178) = 0.21, *p* = .814 nor intervention group *F* (1, 89) = 0.31, *p* = .548, and no significant interaction between intervention group and assessment period, *F* (2, 178) = 0.64, *p* = .526. Secondary outcomes revealed significant main effects for dementia knowledge (DKAS) for the assessment period, *F* (2, 98) = 8.80, *p* <.001, and intervention group *F* (1, 99) = 4.59, *p* = .035, with significantly higher scores at the 3‐ (mean difference = 1.308, *SE* = .31, *p* <.001, 95% CI .692, 1.924) and 6‐month follow‐up (mean difference = .814, *SE* = .31, *p* = .010, 95% CI .203, 1.425); and significantly higher scores in the intervention compared to control (mean difference = .842, *SE* = .393, *p =* .035, 95% CI .062, 1.621). A significant interaction between assessment period and intervention group *F* (2, 98) = 3.33, *p* = .040 indicated that the increase in scores at the 3‐ and 6‐month periods were greater for the intervention group.

**Conclusions:**

This is the first time a dementia training resource for interpreters has been trialled. Preliminary analyses revealed an improvement in interpreter’s dementia knowledge.

